# Animal organoids as models for integrated One Health research

**DOI:** 10.1016/j.onehlt.2026.101539

**Published:** 2026-08-12

**Authors:** Inés García-Rodríguez, Lucía Barrado-Gil, Covadonga Alonso, Miguel Ángel Cuesta-Geijo

**Affiliations:** Dpto. de Biotecnología, CSIC-INIA, Instituto Nacional de Investigación y Tecnología Agraria y Alimentaria, Ctra. de la Coruña Km 7.5, 28040 Madrid, Spain

**Keywords:** Animal organoids, One health, Disease modeling, Antimicrobial resistance, Environmental toxicology, Animal breeding, Climate change impact

## Abstract

Emerging infectious diseases, driven by climate change, urbanization, and global wildlife trade, pose significant threats to public health, ecosystems, and biodiversity. The One Health (OH) approach, which emphasizes the interconnectedness of human, animal, and environmental health, is critical to address these challenges. In this context, animal organoids have emerged as innovative *in vitro* models that can advance OH goals. Organoids are 3-dimensional (3D) structures that closely resemble the cellular composition, organization, and function of the organ they mimic. This review outlines the steps to establish organoid systems and provides an overview of the different animal organoid types developed to date and their contribution to OH. We further explore the applications of these new approach methodologies in the context of OH in studying cross-species diseases, zoonotic infections, environmental pollutant, chemical biocides toxicity, antimicrobial resistance, animal breeding, and pandemic preparedness.

## 1 Introduction

The climate crisis and globalization are significantly increasing the risk of emerging diseases. In recent decades, highly pathogenic bacteria or human and animal viruses such as Influenza virus, Zika virus, Ebola virus (EBOV), severe acute respiratory syndrome coronavirus 2 (SARS-CoV-2), or African swine fever virus (ASFV) have illustrated the profound impact these pathogens can have on global public health, with fatal economic consequences and impact on ecosystems and biodiversity. This rising threat highlights the importance of a **One Health** (OH) approach, which recognizes the interconnectedness of human, animal, and environmental health. In recent years, the concept of OH has gained global attention in our society and focused stakeholders' interest [1]. The latest definition of OH, as described by the One Health High-Level Expert Panel (OHHLEP), is “an integrated, unifying approach that aims to sustainably balance and optimize the health of people, animals, and ecosystems. It recognizes the health of humans, domestic and wild animals, plants, and the wider environment (including ecosystems) are closely linked and interdependent” [2]. To strengthen outbreak preparedness in this new era of infectious disease, innovative strategies are urgently needed. Among them, the development of physiologically relevant laboratory models should be prioritized to enhance our understanding of disease pathophysiology and to accelerate the discovery of effective therapeutics that could reduce the global burden of disease.

**Fig. 1:**
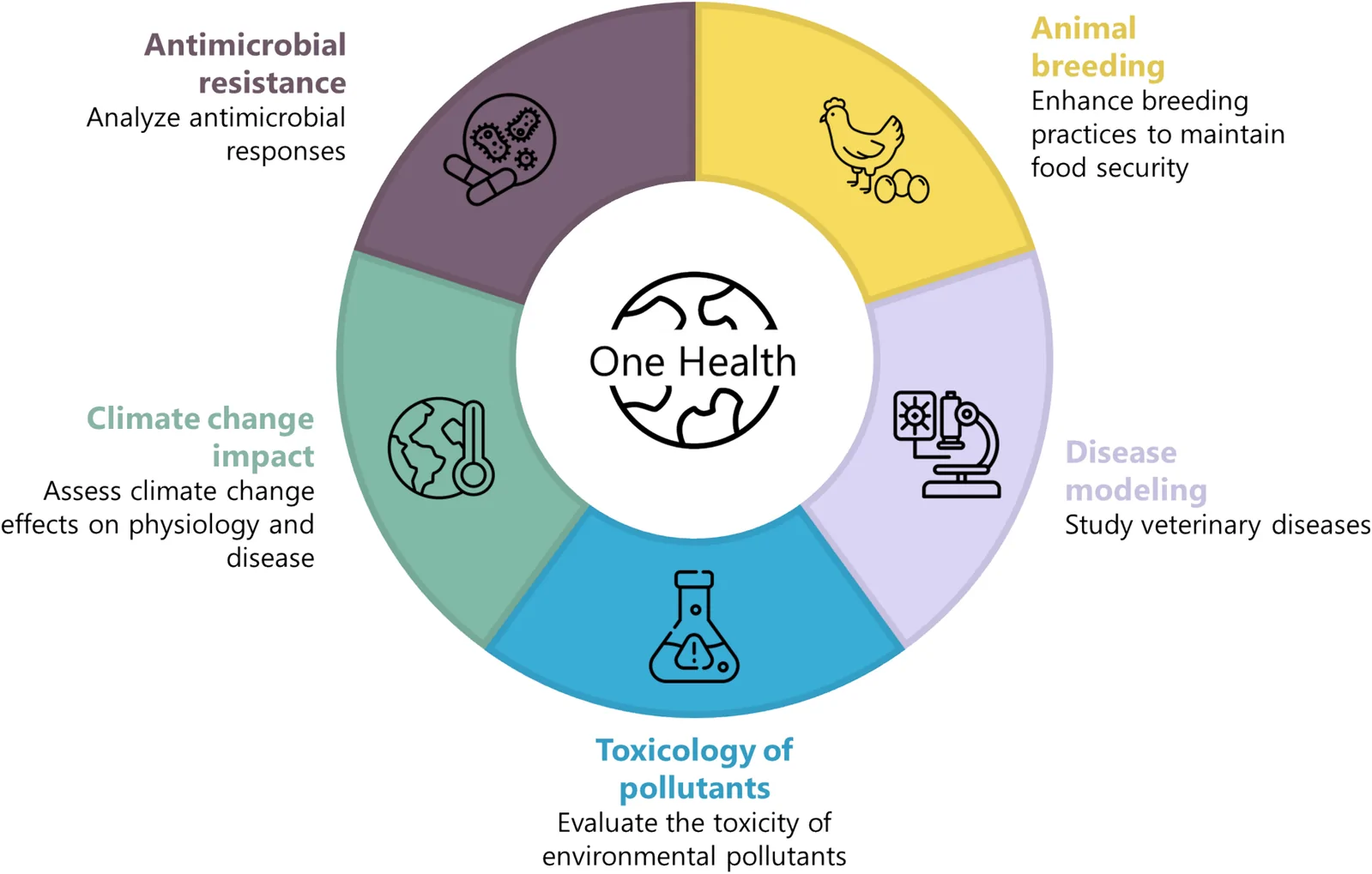
Applicability of animal organoids in different fields of research within the One Health concept.

Within this framework, the development and use of animal organoids offer a valuable tool in advancing OH goals. These organoids can enhance disease modeling, improve our understanding of zoonotic infections, study the toxicology of environmental pollutants, help combat antimicrobial resistance (AMR) by analyzing antimicrobial responses, aid in animal breeding, and help assess the impact of climate change on physiology and disease in animals (Fig. 1). As complementary experimental models, they can reduce reliance on animal experimentation, making them particularly valuable for integrated health research.

Organoids are *in vitro* three-dimensional (3D) structures of self-organized cells that closely recapitulate the architecture and function of the organ they mimic [3]. This innovative culture technique addresses several limitations of traditional immortalized cell lines. While immortalized cell lines provide a practical and accessible platform, they lack the ability to mimic the complex tissue functions, cellular differentiation, and microenvironment found *in vivo* [4]. Unlike immortalized cell lines, which often originate from tumors or acquire genetic abnormalities *in vitro*, organoids preserve multiple cell types, maintain tissue architecture, and sustain crucial cell-cell interactions [5]. Although organoids can show greater variability between experiments due to donor-to-donor differences, this variability also reflects their ability to better recapitulate interindividual diversity, which can particularly valuable when modeling biological differences across age or sex. Specifically, organoids can be derived from individuals of different ages and sexes allowing researchers to model how biological variables like age-related changes [6] or sex-specific responses [7] impact disease progression and treatment outcomes [8]. In contrast, immortalized cell lines typically exhibit chromosomal abnormalities and mutations that alter their biological behaviour and can change further with ongoing culturing, raising concerns about their physiological relevance [9], [10]. Besides, the results obtained in immortalized cell lines may not always fully align with those observed in the final *in vivo* model, which could lead to some inefficiencies in terms of time and economic resources [11], whereas organoids provide a more physiologically relevant model, as they better recapitulates tissue architecture and cellular diversity than immortalized cell lines.

Historically, animal models have been instrumental in understanding disease and developing medical interventions. However, these models come with significant drawbacks, including ethical concerns, high costs, limited sample sizes, lengthy procedures, and low experimental throughput [12]. Although current organoid technologies still rely, in many cases, on animal-derived components, such as extracellular matrices (*e.g.*, Matrigel®) and serum-containing supplements, the field is rapidly advancing towards the development of defined animal-free systems, including synthetic or recombinant extracellular matrices and serum-free culture media. In addition, once established, organoid cultures can be expanded and maintained to generate large amounts of experimental material from a limited number of donor animals, enabling multiple mechanistic, screening, and preclinical studies that would otherwise require substantially higher numbers of experimental animals.

Organoids offer sophisticated complementary models that bridges some of these gaps. They can be maintained long-term and are compatible with cutting-edge molecular techniques such as genetic editing, live-cell imaging, transcriptomic profiling, and epigenetic analyses. Although these molecular techniques can also be applied to immortalized cell lines, organoids provide additional biological complexity and cellular heterogeneity, making them versatile tools for biomedical research offering higher throughput potential, while contributing to the Reduction principle of the 3Rs.

**Fig. 2:**
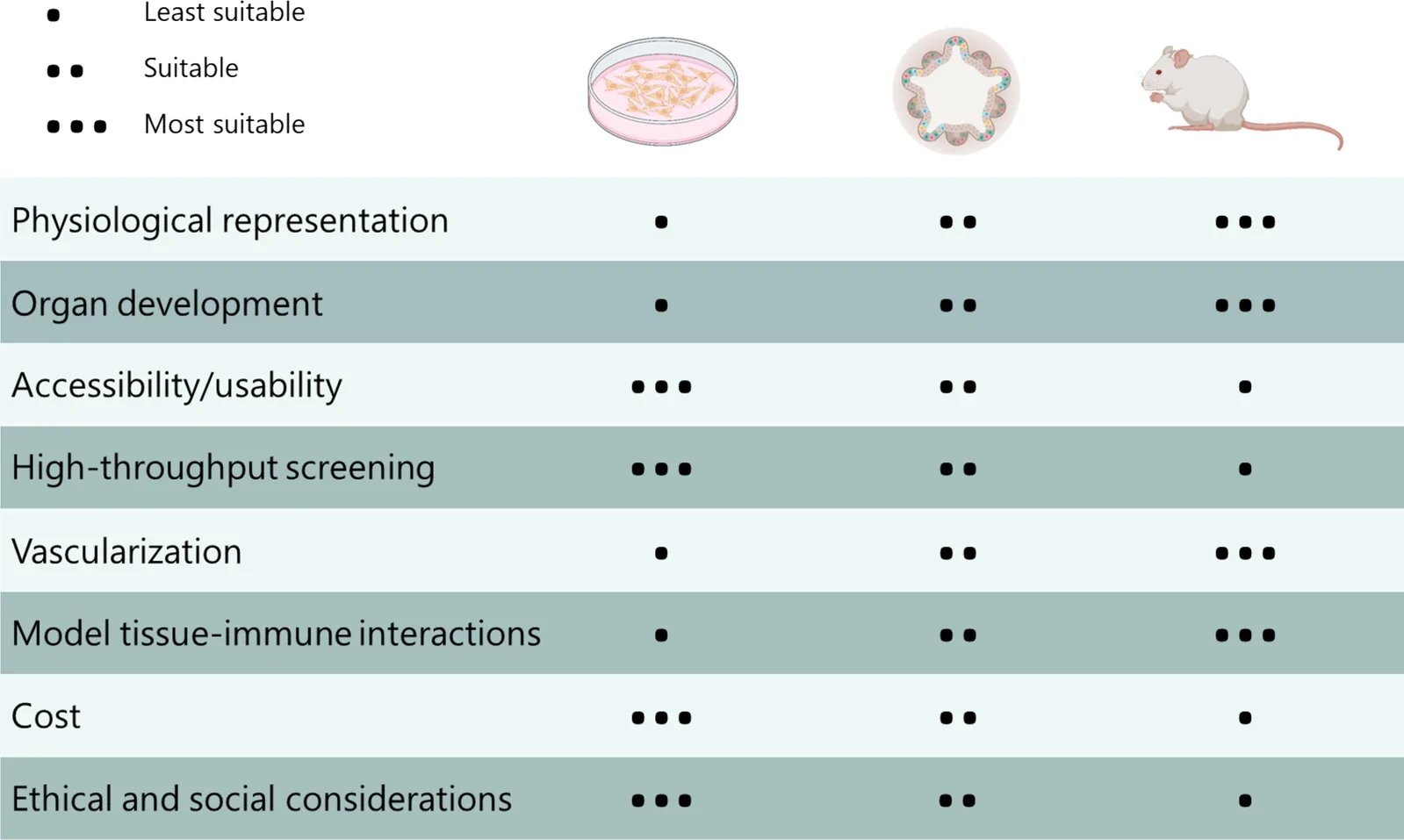
Comparison of organoids with already established model systems, as defined by immortalized cell lines and animal models in general. The score tags can be defined as least suitable (one dot), suitable (two dots), and most suitable (three dots).

A comparative among organoids, immortalized cell lines, and animal models can be found in Fig. 2.

Despite significant advancements in human organoid research, the development of animal organoids remains comparatively limited. Most early breakthroughs were achieved using mice [13], [14], whose organoid systems were initially developed and served as foundational models for the subsequent development of human organoids. In this review, we will outline the general steps to start generating animal organoids, examine the current developments of organoid research in a wider range of animal species, and evaluate their contributions to the advance of OH.

## 2 Starting research with animal organoids

The development of animal organoids begins with the selection of an appropriate cell source. Organoids can be derived from adult stem cells (ASCs), embryonic stem cells (ESCs), or induced pluripotent stem cells (iPSCs) [15]. ASC-derived organoids depend of specialized media supplemented with growth factors that sustain tissue homeostasis [3], [13]. In contrast, ESC/iPSC-derived organoids are produced through stepwise differentiation protocols designed to recapitulate the signalling events that occur during organogenesis [3], [14].

The generation of ASC-derived organoids requires access to primary tissue samples, but these organoids typically retain adult tissue characteristics, making them highly representative of the tissue of origin [16]. Most protocols for animal species organoid culture are adapted from methods established for human or mouse models. Generally, the process begins with enzymatic dissociation of tissue or biopsy samples to isolate its resident stem cells [17]. The isolated cells are then embedded in a supportive matrix, often Matrigel®, to enable 3D growth and organization [18]. To mimic the specific organ, tailored media with specific growth factors are used. For instance, gut organoid culture medium contains compounds that stimulate the Wingless and INT-1 (Wnt) signalling pathway, which is essential for crypt maintenance and spatial patterning [13], [17]. In contrast, airway organoid cultures rely on factors such as FGF7 or FGF10 to stimulate the Fibroblast Growth Factor Receptor 2b (FGFR2b) signalling pathway, which is crucial to maintain epithelial integrity and drive differentiation into the various airway cell lineages [19].

ESC/iPSC-derived organoids offer the advantage of enabling the generation of multiple organ types from a single cell source, although they frequently exhibit a fetal-like phenotype [16]. iPSCs have been established for several animal species, including pigs, cattle, horses, dogs, and chickens [20]. Despite this, their use in animal organoid research remains limited. Notable examples include cerebral organoids from the endangered rhinoceros *Dicerorhinus sumatrensis* [21] and retinal organoids from rhesus macaques [22]. A comprehensive summary of organoids developed across different species and their applications is presented in Table 1.

**Table 1:** Overview of nonrodent animal-derived organoid models from different species and classified based on their main application within the OH scope.

Animal	Organoid model	Applications	Reference
Pig	Esophageal submucosal gland	Model development	[23]
	Intestine	Model development	[24], [25], [26], [27], [28], [29], [30], [31], [32], [33]
		Disease modeling	[34], [35], [36], [37], [38], [39], [40], [41], [42], [43], [44], [45]
		Study of epithelial regeneration	[46]
		Comparative with original tissue	[47]
		Innate immune response studies	[48]
		Toxicity of compounds	[49]
		Response to heat stress	[50]
		Response to dietary compounds	[51], [52], [53], [54], [55]
	Gallbladder	Disease modeling	[56]
	Testis	Model development	[57], [58], [59]
		Toxicity of compounds	[60], [61]
	Airway	Model development	[62]
		Disease modeling	[63], [64], [65], [66], [67], [68], [69]
		Interaction with microbiota	[70]
	Endometrium	Model development	[71]
	Oviduct	Model development	[72]
	Mammary gland	Model development	[73]
Dog	Intestine	Model development	[24], [74], [75], [76], [77], [78], [79]
		Disease modeling	[80]
		Drug permeability	[81]
	Tumor	Model development	[82], [83], [84], [85], [86]
		Drug response	[87], [88], [89], [90]
	Cornea	Model development	[91]
	Liver	Model development	[79]
		Disease modeling	[92], [93], [94]
	Mammary gland	Model development	[82], [95]
	Airway	Model development	[83]
	Oviduct	Model development	[72]
	Skin	Model development	[96], [97]
	Kidney	Model development	[98]
	Pituitary gland	Model development	[86]
Cow	Intestine	Model development	[24], [99], [100], [101], [102], [103], [104], [105]
		Disease modeling	[41], [106], [107], [108]
		Response to food contaminant	[109]
	Airway	Model development	[110]
		Disease modeling	[111], [112], [113], [114]
	Mammary gland	Model development	[115]
	Oviduct	Model development	[72]
		Response to heat stress	[116]
	Ovary	Model development	[117]
	Testis	Model development	[118]
	Gallbladder	Model development	[119]
Chicken	Intestine	Model development	[24], [32], [120], [121], [122], [123], [124], [125], [126], [127], [128]
		Disease modeling	[129], [130], [131]
		Response to probiotics	[132]
		Response to dietary compounds	[129]
		Response to food contaminant	[133]
Cat	Intestine	Model development	[24]
		Disease modeling	[134], [135]
	Liver	Disease modeling	[136]
		Drug response	[137]
	Oviduct	Model development	[72], [138]
	Tumor	Drug response	[87]
	Cornea	Model development	[91]
	Mammary gland	Model development	[73]
	Endometrium	Toxicity of compounds	[139]
Horse	Intestine	Model development	[24], [140], [141], [142]
		Disease modeling	[143]
	Oviduct	Model development	[72], [144]
	Endometrium	Model development	[145], [146], [147]
		Treatment development	[148]
	Mammary gland	Model development	[73]
Non-human primate	Testis	Model development	[60]
	Intestine	Model development	[30], [149]
	Retina	Comparison to human organoids	[22]
	Airway	Drug response	[150]
	Liver	Drug response	[150]
	Kidney	Drug response	[150]
	Pancreas	Model development	[151]
Sheep	Intestine	Model development	[24]
		Disease modeling	[152]
		Response to food contaminant	[153]
		Toxicity of compounds	[154]
	Pancreas	Model development	[155]
	Rumen	Model development	[156]
	Mammary gland	Model development	[157]
Bat	Intestine	Viral susceptibility	[158], [159], [160], [161], [162], [163]
	Airway	Viral susceptibility	[161], [162], [164], [165]
	Kidney	Viral susceptibility	[162]
Rabbit	Intestine	Model development	[166]
		Disease modeling	[167]
	Mammary gland	Model development	[73]
	Liver	Disease modeling	[168], [169]
Goat	Mammary gland	Disease modeling	[170]
		Lactation process studies	[171]
Camel	Airway	Viral susceptibility	[172], [173]
Fish	Retina^a^	Development studies	[174]
	Rectal gland^b^	Model development	[175]
Deer	Mammary gland^c^	Model development	[73]
Snake	Venom gland^d^	Secretion of active toxins	[176]
Rhinoceros	Brain	Model development	[21]

a *Oryzias latipe* and *Danio rerio*.

b *Squalus acanthias*.

c *Odocoileus virginianus*.

d *Naja pallida*, *Naja annulifera*, *Naja nivea*, *Naja atra*, *Aspidelaps lubricus cowlesi*, *Echis ocellatus*, *Deinagkistrodon acutus*, *Crotalus atrox*, and *Bitis arietans*.

## 3 Disease modeling

### 3.1 Infectious diseases

The major advancements in animal organoids for disease modeling have focused on infectious diseases [177]. These diseases are caused by pathogens including bacteria, viruses, parasites, or fungi, impacting both animal health and productivity [178], and, in many cases, public health through their potential for cross-species transmission.

Multiple infectious diseases have been studied using organoids from livestock and domestic animals. These studies include the development of porcine airway organoids to study infection of porcine respiratory coronavirus where the innate immune response upon infection was characterized [63]. A similar model has also been used to explore the pathogenesis of different swine influenza A viruses [64], [179]. Additionally, several models of porcine enteroids have been developed to further understand the infection of porcine epidemic diarrhea virus (PEDV) [36], [37], [45], porcine deltacoronavirus [38], [39], transmissible gastroenteritis virus (TGEV) [42], and *Lawsonia intracellularis* [54]. Other examples include the development of caprine mammary gland organoids to study caprine arthristis encephalitis virus (CAEV) [170], feline enteroids to study feline coronaviruses [135], rabbit enteroids to examine rabbit calicivirus, [167] chicken enteroids to study influenza viruses [131], [165] and *Eimeria tenella* [131], or bovine enteroids for the infection of rotavirus [107].

**Fig. 3:**
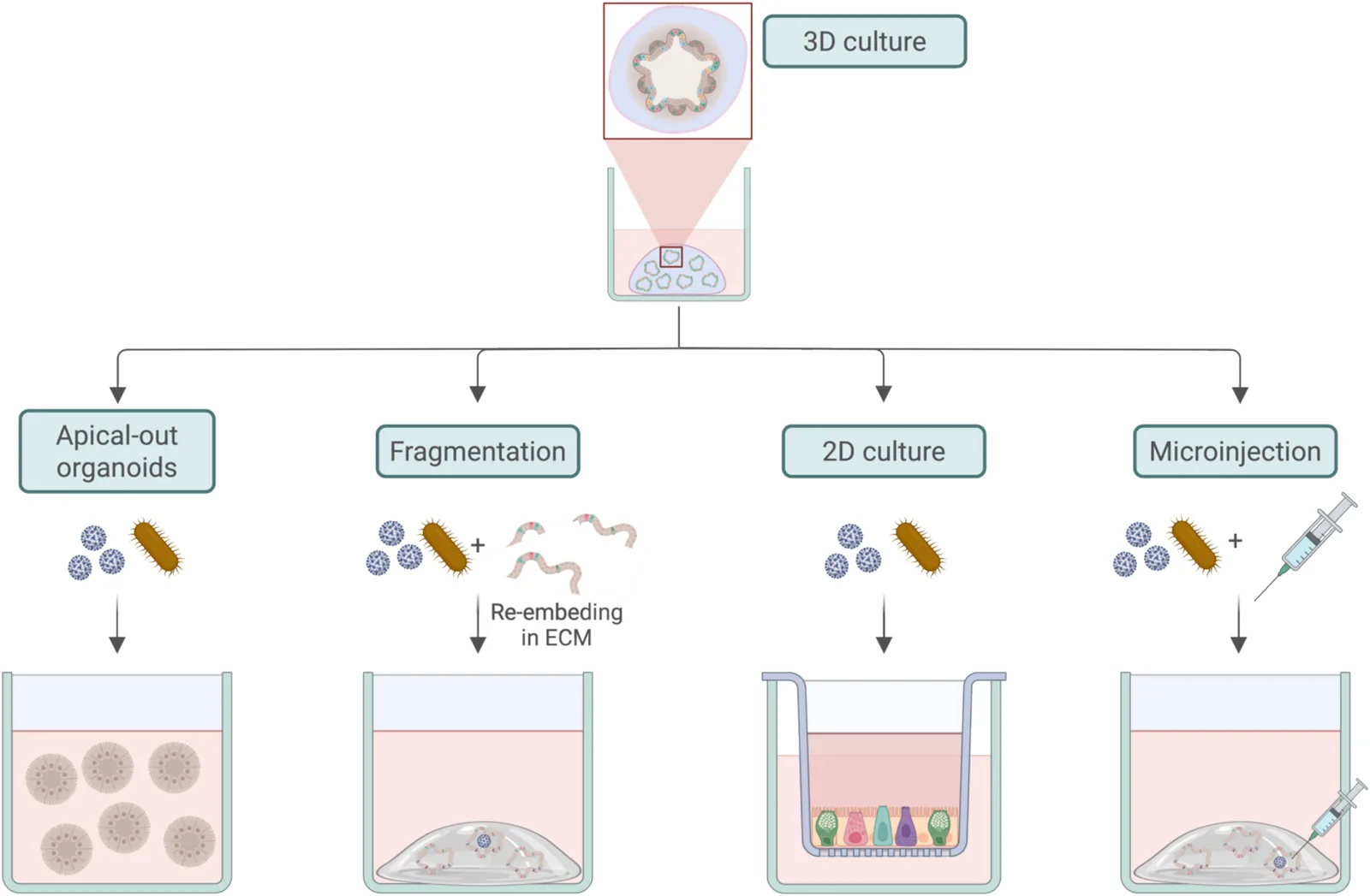
Schematic representation of the different adaptations that can be made from organoids to enhance their utility for the study of infectious diseases.

Organoid models are by definition 3D structures that are typically either embedded in an ECM gel or suspended freely in culture medium [180]. A key factor in modeling infections in organoids is determining whether the pathogen enters the cell through the apical or the basolateral side due to orientation. Several adaptations developed for human organoid models can be applied to animal-derived organoids to enhance their use in infectious disease research. In 3D organoids, the apical surface of the cells is usually oriented towards the lumen, which limits direct access of the pathogen. To overcome this, various strategies have been developed (Fig. 3). These include the generation of inverted polarity or “apical-out” organoids in 3D culture [181], [182], fragmentation of the organoids for infection followed by re-embedding in ECM [183], and culturing organoid-derived monolayers on Transwell inserts, which allows access to both the apical and the basolateral surfaces [184], [185], [186]. Alternatively, the apical side can be accessed through direct microinjection of the organoids, which preserves their original structure, but this process is labor-intensive and technically demanding [19], [187], [188]. Applying some of these adaptations to animal organoids can significantly expand their utility in studying host-microbe interactions.

Once the organoid model has been selected, researchers commonly utilize imaging, sequencing, and pathogens production measurements. These approaches allow for the analysis of pathogen replication dynamics and cytopathic effects (CPE) in a way that reflects tissue-specific characteristics and microenvironmental influences, enabling the study of particular routes of infection or metabolic activity, which are not accessible on immortalized cell lines [159].

Beyond studying infections by single pathogens, organoids serve as powerful tools to investigate co-infections involving multiple microbes. To date, research on co-infections has primarily utilized human organoid models, but the methods can be applied to animal organoids. For example, ectocervical organoids have been used to examine co-infection dynamics of Human papillomavirus (HPV)16 E6E7 and *Chlamydia trachomatis*. The results showed that HPV16 E6E7 slows down *C. trachomatis* life cycle, while *C. trachomatis* interferes with HPV-induced mechanisms that preserve cellular and genome integrity [189]. Similarly, mouse intestinal organoids revealed that *Lactobacillus acidophilus* alleviates the damage caused by *Salmonella typhimurium* infection [190]. In human skin organoids, *Cutibacterium acnes* was shown to protect against the effects of *Staphylococcus aureus* infection [191]. Finally, sequential infections on a human airway epithelium model demonstrated that pre-infection with rhinovirus or Influenza A and B viruses inhibits SARS-CoV-2 replication, while SARS-CoV-2 did not affect the replication of these seasonal respiratory viruses [192].

### 3.2 Zoonotic infections and cross-species infections

Zoonotic infectious diseases are responsible for an estimated 60% of emerging human infections [193], thus the study of the infection in their primary or intermediate hosts is key to understand spillover dynamics. In this context, organoids derived from humans and diverse animal hosts provide a valuable experimental platform to compare host susceptibility, tissue tropism, and pathogen adaptation during emerging infections. These models also reduce reliance on costly animal models while facilitating the rapid development and evaluation of effective therapeutic approaches.

The application of animal-derived organoids to study zoonotic viruses has been reviewed elsewhere [194]. In brief, that work highlights how species-specific organoid platforms enable the investigation of viral tropism, virus-host interactions, and cross-species transmission. This is particularly valuable because defining cell type tropism reveals which cells (of the complete epithelium) are permissive to infection and therefore informs of mechanism of viral entry and replication [195].

Zoonotic diseases caused by bacteria and parasites have also been studied using animal organoids. This is the case of toxoplasmosis, a disease usually spread through consumption of undercooked meat infected with the protozoan parasite *Toxoplasma gondii* [196]. Another example is salmonellosis that spreads through infected poultry and livestock carrying *S. typhimurium* [197]. Both of these pathogens can persistently infect the gastrointestinal tract of cattle and pigs. To study these interactions with the species of interest, bovine and porcine enteroids have been developed. Organoids from both species were susceptible to infection, while control murine enteroids did not support the infection of *S. typhimurium* [41]. These findings underscore the importance of species-specific organoid models to accurately investigate host-pathogen interactions.

In addition to reservoir hosts, intermediate hosts also play a critical role in zoonotic disease transmission [195]. Although MERS-CoV and SARS-CoV-1 likely originated in bats, other species such as palm civets and dromedary camels facilitated their transmission to humans [198]. Airway organoids derived from camels have been shown to support MERS-CoV infection [172], [173], but not SARS-CoV-2 [172], suggesting that camels are unlikely to contribute to the spread of the latter virus. Overall, the development of organoids from diverse animal species involved in zoonotic transmission provides a powerful platform to investigate host susceptibility, viral evolution, and disease pathogenesis.

### 3.3 Comparative models of non-infectious diseases

Although infectious diseases constitute a major application of animal organoids in OH research, these models are also increasingly used to investigate naturally occurring veterinary diseases. Animal organoids support the development of treatments that improve animal health while also progressing our understanding of these diseases. Moreover, the data obtained from veterinary diseases may also be translated to human diseases contributing to the OH paradigm [199], [200], as many chronic diseases, including inflammatory disorders and cancer, share biological and molecular mechanisms across species.

One example is the characterization of inflammatory bowel disease (IBD) in canine organoids. IBD is a chronic inflammatory disorder that affects the intestinal tract, and while the exact mechanisms behind the disease remains unclear, there is evidence suggesting similarities between IBD in humans and dogs [201], [202]. Canine enteroids and colonoids (organoids from the intestinal tract) have been obtained from both healthy and diseased animals [74], [75], [77] and they can be employed to characterize disease and as a platform for drug screening without the need of using live animals [74], [177].

Similarly, tumor organoids have been established primarily from companion animals (particularly cats and dogs) [177]. Tumor organoids have been derived from various cancer tissues and have been used as a platform for drug testing showing different sensibilities by type and species [87]. In addition, dog bladder tumor organoids from urine samples have been established, employed for anticancer drug testing, and compared to healthy bladder organoids using RNA sequencing to search for new diagnostic markers [88]. Moreover, because their shared biological features discoveries using these animal organoids can help further comprehend human cancer biology [87], [88], [199].

## 4 Toxicological analysis of environmental pollutants

Organoids offer a valuable platform to evaluate the toxicity of various environmental pollutants. These pollutants can cause biological hazards and are released by nature or produced by industrial activity and human life [203], they include air pollutants, heavy metals, pesticides, or persistent organic pollutants [204]. Intestinal and liver organoids have been shown to functionally express key xenobiotic-metabolizing enzymes such as cytochrome P450 [205]. Organoids are particularly well-suited for toxicological studies because they enable the analysis of chemical effects across different organ systems, support the investigation of developmental toxicities by using organoids at different maturation stages, and can model carcinogenicity through sustained cell proliferation (an essential process for the accumulation of genetic and epigenetic alterations) [204].

To date, direct applications of animal organoids in environmental toxicology remain limited. For example, porcine testicular organoids have been used to evaluate the toxicity of mono(2-ethylhexyl) phthalate (MEHP), a widespread environmental contaminant known to cause endocrine disruption, revealing increased autophagic activity in response to exposure [60]. Similarly, bovine intestinal organoids have been used to assess the toxicity of deoxynivalenol and to evaluate the detoxifying capacity of *Lactobacillus* [109]. In contrast, a wider range of toxicological studies has been conducted using human organoid models, providing a methodological framework that can be readily adapted to animal systems. These include the use of breast organoids to evaluate the development toxicity of cadmium, where physiologically relevant concentrations were found to impair mammary stem cell activity and inhibit branching morphogenesis [206]. Similarly, liver and cardiac organoids have been used to asses toxicity of lead, mercury, thallium, and glyphosate; with cardiac organoids also exhibiting a decreased beating rate upon exposure [207]. Colon organoids have been employed to study the molecular effects of ethanol exposure, identifying several differentially expressed genes consistent with those known to be affected by alcohol, together with an enrichment on pathways related to colon cancer [208]. In another study, neural organoids were used to demonstrate the neurotoxic effects of the pesticide rotenone at low exposure levels, consistent with its classification as a developmental toxicant [209].

Collectively, these human organoid studies illustrate the breadth of toxicological questions that can be addressed with organoid technology and underscore the potential for translating these approaches to animal organoids.

## 5 Analysis of antimicrobial responses

As mentioned previously, animal organoids have been widely used to investigate host-pathogen interactions. Consequently, these systems also provide valuable tools to evaluate antimicrobial therapies in a tissue- and host-specific context. Rather than directly dissecting the molecular basis of antimicrobial resistance, which are often microbe-driven, animal organoids are particularly well suited to compare host responses that may influence bacterial persistence, biofilm formation, and antibiotic treatment outcomes.

In this context, human organoid models have already been employed to test antimicrobial strategies against biofilms. For example, a human epidermis organoid model was developed incorporating biofilms of resistant bacteria, such as robust methicillin-resistant *Staphylococcus aureus* (MRSA) USA300 and *Pseudomonas aeruginosa* PAO1. This model was used to assess the efficacy of antibiofilm compounds and alternative therapeutic strategies [210]. Similarly, a newly developed antimicrobial compound successfully eradicated MRSA biofilms in a human skin organoid [211], highlighting how organoid-based systems can be used to evaluate novel antimicrobial and host-directed therapies in a physiologically relevant setting.

Importantly, AMR is not limited to human medicine, it also represents a major concern in veterinary contexts [212]. Although direct studies of antibiotic testing in animal-derived organoids remain relatively scarce compared to the human literature, emerging research has demonstrated that organoids from farm animals, particularly porcine intestinal and pulmonary models, can effectively recapitulate host-microbe interactions and responses to therapeutic interventions [8], [213]. These findings suggest that animal organoids could become valuable tools for evaluating antimicrobial strategies across species.

In this context, organoid-based assays may include infecting organoids with bacteria followed by antimicrobial treatment and quantification of bacteria, biofilm disruption, epithelial integrity, and cytokine production.

## 6 Animal breeding

Animal organoids can also be used to enhance animal breeding and help maintain food security through two complementary approaches. On the one hand, it is possible to develop reproductive system organoids to improve reproductive success by advancing the understanding of reproductive physiology and pathology [214]. On the other hand, animal organoids can be used to phenotype animals to improve selective breeding by linking genotype to functional traits [215].

Reproductive system organoids have been developed across multiple species to model tissue-specific physiology and disease. For example, equine endometrium organoids derived from both domestic and endangered species have been established and used to assess responses to exogenous hormonal stimulation [145], [146]. In addition, a 3D collagen-based equine endometrium has been developed to further investigate equine uterine physiology [147]. Oviductal organoids have also been generated from several species, including bovine, porcine, equine, feline, and canine models [72]. Notably, bovine oviductal organoids have been applied to investigate the effects of heat stress, reveling the prospective impact of environmental heat stress on oviductal physiology [116]. Endometrial organoids from domestic cats have been used to assess the toxicity of plastic additives that are used in the biomedical field [139]. Similar organoids systems have been established and characterized in pigs [71].

Significant efforts have also focused on the development of testicular organoids. Non-human primate and pig testicular organoids have been described and used both for drug toxicity and development studies [57], [60]. Similarly, bovine testicular organoids capable of producing testosterone have been generated, providing a functional *in vitro* model for male reproductive endocrinology [118].

Beyond reproductive biology, animal organoids hold considerable potential as functional phenotyping tools for selective breeding. By integrating organoid-based phenotypes with known genotypes, it becomes possible to identify animals with desirable traits for breeding programs [215]. Such traits may include resistance to infectious diseases or improved nutrient utilization efficiency [216], both of which can be modelled using organoid systems. Moreover, these organoids will be useful tools to model the impacts of environmental stressors allowing the farming industry to adapt [217].

## 7 Impact of climate change on physiology and disease susceptibility

Climate change affects animal health through direct physiological stress and indirect effects on infectious disease dynamics. Rising temperatures and more frequent heat stress events can impair tissue homeostasis, compromise barrier function, reduce reproductive performance, and increase susceptibility to disease. Animal organoids can be exposed to varying temperatures and used to evaluate the changes in the epithelium. For example, porcine intestinal enteroids have been used to demonstrate that heat stress induces endoplasmic reticulum stress and impairs intestinal stem cell function, leading to compromised epithelial barrier integrity [50]. Similarly, bovine oviductal organoids exposed to heat stress have been analysed using transcriptomics and extracellular vesicle profiling, revealing alterations in genes microRNAs associated with cellular stress responses, homeostasis, and reproductive function [116].

Beyond these direct effects on host physiology, climate change also alters the ecology of pathogens, vectors, and animal hosts. Climate change affects infectious diseases largely through its impact on vector-borne diseases (VBDs). Shifts in temperature, precipitation, and other climate factors can alter the habitat and activity patterns of both vectors and hosts, thereby changing the spread and prevalence of these diseases [218]. VBDs are usually transmitted by hematophagous arthropods including mosquitos, ticks, or fleas, that can transmit malaria, dengue fever, Chikungunya virus, West Nile virus, or Lyme disease [219], [220].

Animal organoids from mammals can be used to address their possible role in the further transmission of these diseases. More importantly, developing organoids that mimic the organs of arthropods, such as mosquitoes and ticks, would represent a significant advancement in understanding the behaviour of pathogens in their original vectors. However, to date, such organoids have not been developed, as the mammalian organoid technology cannot be directly applied to arthropods [221].

While true organoid models for arthropods are still lacking, there has been progress in developing *ex vivo* cultures of arthropod tissues. Gut *ex vivo* cultures of *Aedes aegypti* mosquitoes have been developed to study the infection of several arboviruses for which antiviral drugs were tested [222]. Likewise, *ex vivo* cultures of midgut, salivary glands, and synganglion of *Ixodes scapularis* ticks have been infected with tick-borne flaviviruses [223]. Beyond arthropods, there is also growing interest in developing organoid or organ-like systems for molluscs [224], particularly to study trematode-host interactions. Collectively, these studies suggest that advancing organoid development in the arthropod field could enhance the understanding of VBDs, the design of treatment options, and the creation of control strategies and general preparedness to emerging and remerging diseases.

## 8 Discussion and future perspectives

Animal organoids have emerged as powerful and versatile experimental models that bridge critical gaps between traditional cell cultures and *in vivo* studies while reducing reliance on animal experimentation. This advantage is particularly relevant for wild animal species, where *in vivo* studies are hindered by ethical, logistical, and regulatory considerations.

Organoids derived from animal tissues provide a timely experimental bridge for OH because they enable tissue-resolved, species-specific biology to be studied under controlled and scalable conditions, while remaining closer to *in vivo* physiology than conventional immortalized cell lines [214]. By preserving key epithelial architecture and functional characteristics of native organs, these models help harmonize evidence across species and disciplines, supporting more comparable mechanistic insights that are directly relevant to the interconnectedness between humans, animals, and the environment [225]. We use a OH perspective to highlight why organoids are informative across multiple application domains.

Building on this rationale, the practical contribution of animal organoids to an OH-oriented agenda becomes most apparent when considering how they address recurrent bottlenecks shared across application domains by using immortalized cell lines or *in vivo* models. Their primary application is disease modeling, where organoids provide a physiologically relevant yet experimentally tractable system to investigate species-specific tissue biology and responses in ways that are difficult to achieve in immortalized cell lines and often impractical to scale *in vivo* [8], [199].

From a OH perspective, this naturally extends to zoonotic and cross-species infection questions. Because organoids can be derived from humans as well as relevant animal hosts and meaningful tissue regions, they enable side-by-side assessment of permissiveness, tropism, and epithelial responses under standardized conditions, thereby helping to reduce uncertainty when translating findings across hosts and when prioritizing surveillance or intervention strategies [199], [225].

An especially promising yet largely unexplored approach would be to systematically study pathogen evolution and cross-species adaptation using organoids derived from multiple host species. By infecting organoids derived from humans, wildlife reservoirs, livestock, and companion animals with the same pathogen under similar conditions, we can compare pathogen replication kinetics, tissue tropism, epithelial damage, and innate immune responses across host species. Additionally, serial passage experiments could be used to model evolutionary trajectory of a pathogen during host switching. This approach could uncover pathogen genetic changes associated with adaptation, altered virulence, or expanded host range. Recent work illustrates the feasibility of this strategy: a panel of airway organoids derived from different animal species was used to analyze the susceptibility to Influenza A virus, detecting host-specific differences in infection rates and cytopathogenicity [226]. Despite the strong theoretical potential of organoids to model this interspecies pathogen mutation and adaptation, there is a near-complete absence of studies comparing how the same pathogen evolves, accumulates mutations, and adapts in organoids from different species. This gap is highly relevant in a OH context, where understanding viral behaviour in alternative or intermediate hosts is essential for anticipating zoonotic risk, spillover events, and host jumps. Leveraging multi-species organoid platforms could therefore provide unprecedented insight into the mechanisms that govern viral host adaptation and emergence [194].

Beyond disease modeling, toxicological analysis represents a second OH-relevant axis in which animal organoids can be particularly informative for species-specific risk assessment. Organoid systems allow adverse effects to be quantified directly at the tissue level under different circumstances such as long or repeated exposures, supporting a more mechanistic and potentially earlier evaluation of hazard than is typical of simplified cell models, while also offering an opportunity to reduce reliance on resource-intensive *in vivo* experiments [205]. Importantly, without organoids, toxicological assessment frequently faces a trade-off between throughput (immortalized cell lines) and physiological relevance (*in vivo*), which can delay decision-making and obscure tissue-specific liabilities.

Apart from toxicological analysis, a closely related field of research is antibiotic and antimicrobial testing. Within this framework, organoids allow an integrated evaluation of antimicrobial activity together with host-relevant readouts, enabling the distinction between true antimicrobial efficacy and epithelial toxicity and allowing investigation of context-dependent effects such as epithelial barrier disruption or restoration [227], [228]. Moreover, cross-species studies can be made with organoids from several animals and humans, which may facilitate drug repurposing and improve the low success rate of candidates reaching clinical use [200]. By contrast, in the absence of organoids, antimicrobial evaluation often focuses primarily on pathogen readouts and fails to capture host tissue effects that are essential for translational relevance, especially across different animal species and production contexts.

Animal breeding and selection provide a further, less frequently emphasized opportunity: organoids can serve as a functional *in vitro* layer between genotype and phenotype. By enabling standardized stimulation and phenotyping of tissue-specific responses (for instance, epithelial resilience, regenerative capacity, or responses to microbial or nutritional cues), organoids may help reveal biological variability that is hidden by environmental and management factors in whole-animal studies [215]. Without organoid-based functional assays, relevant traits often require long, expensive *in vivo* trials and remain difficult to interrogate mechanistically, slowing the translation of biological insights into effective selection strategies.

Finally, the OH value of animal organoids can be framed in the context of environmental pressures, including climate-associated stressors that influence disease susceptibility. Although this area is still emerging, organoids are well suited to study how defined stressors (*e.g.*, heat stress, dietary changes, or co-exposures) affect tissue function and host responses in a species- and tissue-specific manner. Without these systems, the impact of environmental factors on disease risk is often based on observational or animal studies, where causality and mechanism are harder to establish.

Despite the growing use of animal organoids, few studies integrate these models with veterinary clinical validation, reducing its translational value in veterinary medicine. While several human studies have demonstrated correlations between organoid responses and clinical outcomes [229], equivalent evidence in animal health remains extremely limited [202], [228]. This represents a missed OH opportunity, particularly given the relevance of livestock and companion animal diseases to public health, food security, and zoonotic risk.

To date, most animal organoid models have been developed from mammalian species and from other classes, including avian intestinal organoids [129], [130], snake venom gland organoids [176], and fish retinal organoids [174], although such examples remain comparatively rare. Expanding organoid development across a broader range of taxa will significantly enhance the utility of these systems for OH research. Achieving this goal will require deeper insights into organ development and stem cell biology across diverse species [221].

Despite their potential, current organoid systems face significant limitations. Limited scalability, reliance on manual handling, batch-to-batch variability, and the necessity for 3D culture conditions reduce their compatibility with high-throughput and automated platforms [230]. Reproducibility and replicability between laboratories are still ongoing challenges in the organoid field. To address these issues, new standards are being promoted, including greater transparency in reporting, harmonization of protocols [231], and systemic monitoring of phenotypic stability in long-term cultures [16].

In parallel, increasing tissue complexity through the incorporation of vascular and immune components is critical to improve physiological relevance. Vascularization supports oxygen and nutrient delivery, while flow-induced shear stress promotes cell differentiation, maturation, and tissue organization [232]. It is particularly critical for large and metabolically demanding organoids, such as brain organoids, which frequently develop necrotic cores due to limited diffusion [233]. Organ-on-a-chip and microfluidic systems offer promising strategies to introduce controlled vascularization and physiological flow [234]. Similarly, the inclusion of immune cells is necessary to more accurately model host-pathogen interactions, inflammation, and immune-mediated diseases [235]. Although organoids primarily represent the epithelial compartment, they retain the ability to mount intrinsic innate immune responses, including the productions of cytokines and chemokines. However, they still fail to fully recapitulate important immune-mediated processes, including immune cell recruitment, antigen presentation, cytokine amplification, or immune mediated tissue damage. For this reason, immune-competent organoids and co-culture systems are increasingly being developed to better reconstruct tissue complexity and broaden the utility of organoids [236].

## 9 Conclusion

In conclusion, animal organoids can play a central role in advancing the OH paradigm, and constitute more predictive and reproducible models to study cross-species disease dynamics, support drug discovery and repurposing, contribute to the farming industry, and strengthen pandemic preparedness. Although human and murine organoids are well established, a wide range of organoids from livestock, sylvatic species, and exotic animals have been developed over the past decade. While still comparatively limited in number, these emerging models are expanding *in vitro* research to species that were previously inaccessible, opening new opportunities by leveraging unique animal adaptations that can address shared challenges in veterinary and human medicine [199].The development of new organoid models from diverse species holds great potential to address emerging health threats. Looking ahead, broader model development and better standardization across species will be key to fully harness the potential of animal organoids in biomedical research and disease preparedness.

## CRediT authorship contribution statement

**Inés García-Rodríguez:** Writing – review & editing, Writing – original draft, Conceptualization. **Lucía Barrado-Gil:** Writing – review & editing. **Covadonga Alonso:** Writing – review & editing, Conceptualization. **Miguel Ángel Cuesta-Geijo:** Writing – review & editing, Writing – original draft, Funding acquisition, Conceptualization.

## Declaration of competing interest

The authors declare that they have no known competing financial interests or personal relationships that could have appeared to influence the work reported in this paper.

## Data Availability

No data was used for the research described in the article.
